# Glycan-immobilized dual-channel field effect transistor biosensor for the rapid identification of pandemic influenza viral particles

**DOI:** 10.1038/s41598-019-48076-6

**Published:** 2019-08-12

**Authors:** Sho Hideshima, Hiroki Hayashi, Hiroshi Hinou, Shunsuke Nambuya, Shigeki Kuroiwa, Takuya Nakanishi, Toshiyuki Momma, Shin-Ichiro Nishimura, Yoshihiro Sakoda, Tetsuya Osaka

**Affiliations:** 1 Research Organization for Nano & Life Innovation, Waseda University, 513 Waseda-tsurumaki-cho, Shinjuku-ku Tokyo, 162-0041 Japan; 2 Graduate School of Advanced Science and Engineering, Waseda University, 3-4-1 Okubo, Shinjuku-ku Tokyo, 169-8555 Japan; 3 Faculty of Advanced Life Science, Hokkaido University, N21, W11, Kita-ku, Sapporo-shi 001-0021 Hokkaido, Japan; 4 Faculty of Veterinary Medicine, Hokkaido University, N18, W9, Kita-ku, Sapporo-shi 060-0818 Hokkaido, Japan; 5 Present Address: Research Initiative for Supra-Materials, Interdisciplinary Cluster for Cutting Edge, Shinshu University, 3-15-1 Tokida, Ueda-shi Nagano, 386-8567 Japan

**Keywords:** Biosensors, Nanosensors

## Abstract

Pandemic influenza, triggered by the mutation of a highly pathogenic avian influenza virus (IFV), has caused considerable damage to public health. In order to identify such pandemic IFVs, antibodies that specifically recognize viral surface proteins have been widely used. However, since the analysis of a newly discovered virus is time consuming, this delays the availability of suitable detection antibodies, making this approach unsuitable for the early identification of pandemic IFVs. Here we propose a label-free semiconductor-based biosensor functionalized with sialic-acid-containing glycans for the rapid identification of the pandemic IFVs present in biological fluids. Specific glycans are able to recognize wild-type human and avian IFVs, suggesting that they are useful in discovering pandemic IFVs at the early stages of an outbreak. We successfully demonstrated that a dual-channel integrated FET biosensing system, which were modified with 6′-sialyllactose and 3′-sialyllactose for each gate area, can directly and specifically detect human H1N1 and avian H5N1 IFV particles, respectively, present in nasal mucus. Furthermore, to examine the possibility of identifying pandemic IFVs, the signal attributed to the detection of Newcastle disease virus (NDV) particles, which was selected as a prime model of a pandemic IFV, was clearly observed from both sensing gates. Our findings suggest that the proposed glycan-immobilized sensing system could be useful in identifying new pandemic IFVs at the source of an outbreak.

## Introduction

Pathogenic viruses present in the environment, such as influenza and Ebola, are very harmful to public health. In particular, influenza virus (IFV) has threatened human life many times in history as a result of pandemic outbreaks^1,2^. A pandemic will occur when avian IFVs of low pathogenicity evolve to become highly pathogenic as result of mutation. It has recently been reported that highly pathogenic H7N9 or H5N1 viruses have the potential to trigger the next pandemic, and should be carefully monitored^3–5^. When pandemic IFVs appear and acquire human-to-human transmissibility, they are thought to become able to bind not only to the avian glycan receptor but also to the human glycan receptor on human epithelial cells^5^. Therefore, glycans can be used to discover new pandemic IFVs at the early stages of an outbreak.

**Figure 1:**
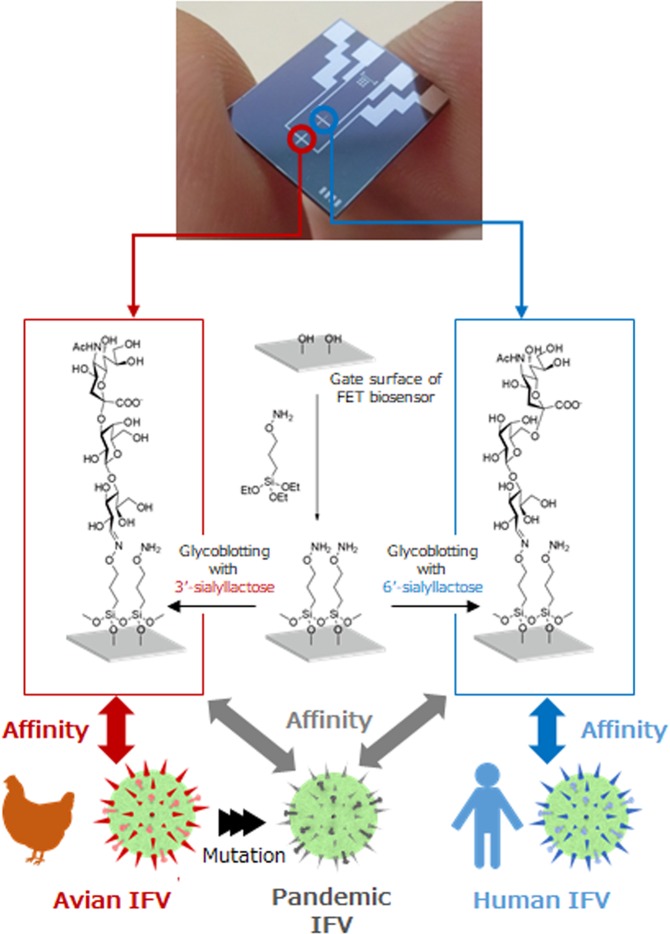
Schematic illustration of the identification of pandemic influenza viral particles by using glycan-immobilized dual-channel field effect transistor biosensor.

Rapid detection of mutations in avian IFVs minimizes the damage caused by pandemic influenza. Since the detection of pandemic IFVs at the source of the influenza outbreak is essential, a simple and sensitive detection method that can be used in multiple locations in the field prior to more precise analyses of the virus such as its gene sequences and its hemagglutinin (HA) subtype, is required to be developed. Traditional detection methods, such as polymerase chain reaction (PCR) or immunochromatography, have drawbacks. The PCR method, although it has extremely high sensitivity, is difficult to use outside of a standard laboratory setting because reagents and expensive instruments are required^6^. Immunochromatography is convenient, but its detection ability is insufficient in the early stages of viral mutation because it needs specific antibodies that are obtained by analyzing pandemic IFVs, and its assessment of subtypes requires a subjective visual observation^7^. As an alternative to these conventional analytical methods, a field effect transistor (FET) biosensor can provide high sensitivity, combined with simple operation, because it can directly detect the intrinsic charge of its targets^8,9^, which means that the sensor has its potential not only to detect easily proteins or monitor cell activities^10,11^ but also to sequence genomes^12^ in the medical and healthcare fields. Furthermore, the FET biosensors, which are miniaturized and integrated by using semiconductor processes, would be cost-competitive in their full-scale practical applications. In our previous work, we have proposed glycan-immobilized FET biosensors that can detect and discriminate between human and avian IFV HA at attomolar levels^13^. These findings are based on the IFV infection mechanism whereby the virus initiates its infection through its surface glycoproteins recognizing sialic acid-terminated glycans which exist on human epithelial cells^14^. Trisaccharides terminating in sialic acid-α2,6-galactose (6′-sialyllactose, hereafter denoted as Siaα2,6′Lac), and in sialic acid-α2,3-galactose (3′-sialyllactose, hereafter denoted as Siaα2,3′Lac), mainly recognize human and avian IFV HA, respectively. The proposed host cell surface-mimetic glycan-immobilized platform has so far been adopted to detect IFV using potentiometric or impedance biosensors^15,16^. Additionally, by modifying separated surface area with Siaα2,6′Lac and Siaα2,3′Lac, the combination of the dual glycan-immobilized surfaces is expected to acquire ability to discover the new pandemic IFVs; the mutant pandemic IFV adsorbs onto both of the dual glycan-immobilized surfaces, while the wild avian IFV preferably adsorbs only onto Siaα2,3′Lac-immobilized surface (Fig. 1).

To achieve a direct detection of IFVs present in mucus samples on site, *e.g*. poultry farms, the sensing surface of the biosensor needs to directly capture IFV particles in biological fluids collected from the hosts, such as nasal mucus. However, a major component of mucus, namely mucin, increases the viscosity of biological fluids as a result of its agglutination^17^, and this high viscosity might affect the diffusion of IFVs to the surface of the biosensor. The mucoactive agent, l-cysteine ethyl ester (LCEE), is known to cleave disulfide bonds between mucin molecules^18^, suggesting that its use in the detection procedures will enhance signal generation due to a reduction in viscosity of biological fluids to be assayed. In addition, a portable biosensing system, which can be operated using the low-level electric power provided by a smartphone, will allow users to easily obtain and share sensing data, expecting that it could be useful for border control against potential intrusion of pandemic IFVs.

In this study, we demonstrated the usefulness of glycan-immobilized dual-channel FET biosensors in the early identification of pandemic IFVs in mucus samples. The ability to detect whole viral particles present in a biological sample, for example sample of nasal mucus as well as to quantitatively detect human and avian IFVs by each of the glycan-immobilized FETs were evaluated. The effect of the viscosity of the mucus sample on the direct detection of IFV particles was also examined. Furthermore, we assessed the feasibility of portable biosensing via a Bluetooth connection, so that the FET-based biosensing system, controlled by a smartphone device, could be used anytime and anywhere. Additionally, by reducing the viscosity of the sample using the LECC reagent, the utility of the glycan-immobilized FET biosensor for the specific detection of human IFV, avian IFV, and Newcastle disease virus (NDV) particles in nasal mucus was demonstrated. It should be noted that the NDV was used as a prime model of pandemic IFVs, because the NDV, as well as pandemic IFVs, possesses a viral surface protein which recognizes both α2,6 and α2,3 sialic acid-linkages^19,20^, suggesting that the glycan-immobilized dual-channel FET biosensors could be used as a detection method to find new pandemic IFVs at the early stages of an outbreak.

## Results and Discussion

### Direct detection of influenza virus by a glycan-immobilized FET biosensor

**Figure 2:**
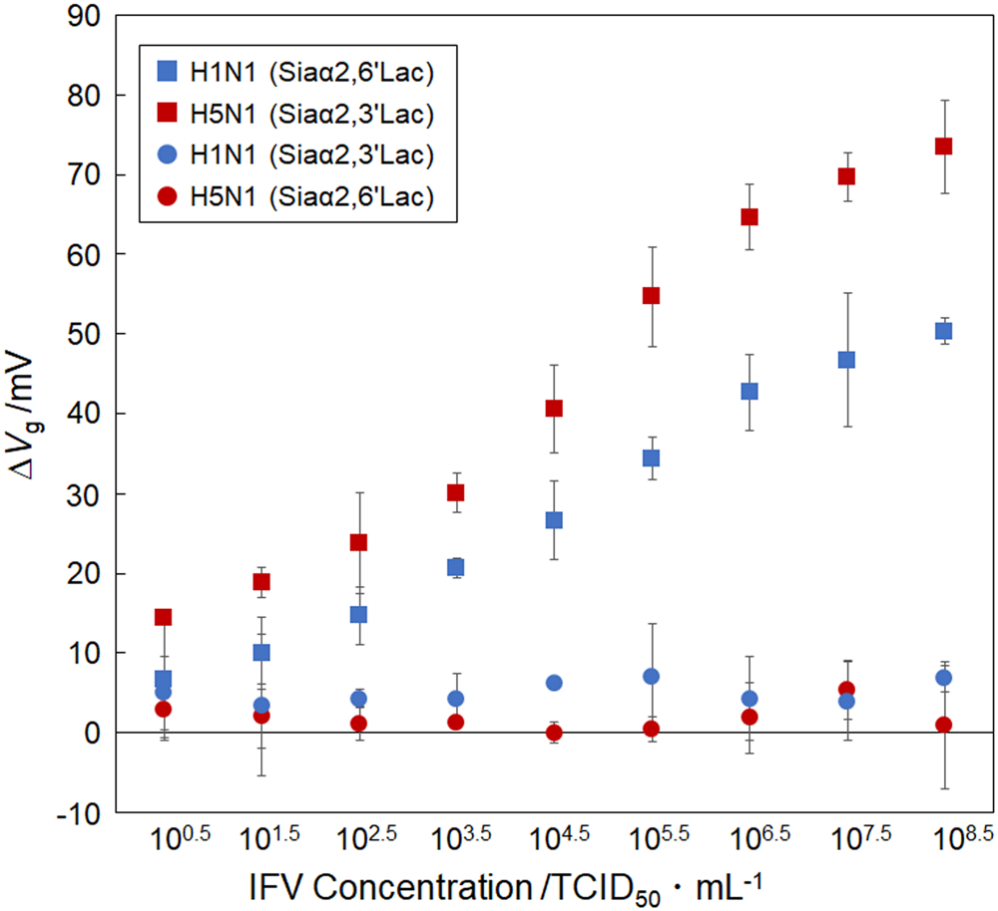
Quantitative determination of influenza virus (IFV) suspended in PBS using glycan-immobilized FET biosensors. Detection of human H1N1 IFV and avian H5N1 IFV at concentrations ranging from 10^0.5^ to 10^8.5^ TCID_50_/mL (50% tissue culture infectious dose) using either a Siaα2,6′Lac-immobilized FET or a Siaα2,3′Lac-immobilized FET. Error bars represent the standard deviation (N = 3).

To confirm the specificity of the FET biosensor for different IFV subtypes, we measured the electrical response (Δ*V*_g_) following the addition of IFVs to an FET with a glycan immobilized on its surface. For the FET biosensor with the Siaα2,6′Lac-immobilized surface, a Δ*V*_g_ (50 mV) was observed when the H1N1 IFV (10^8.5^ TCID_50_/mL) was added. The shift in the gate voltage in a positive direction was caused by the adsorption of the negatively charged IFV (pI = 4–4.5^21^) in 1 × PBS (pH 7.4). On the other hand, when H5N1 avian IFV was added to the FET with the Siaα2,6′Lac-immobilized surface, no significant FET response was observed because non-specific adsorption of the avian IFVs was suppressed due to the hydrophilicity of the glycan. Atomic force microscope (AFM) images of the Siaα2,6′Lac-immobilized surface after the addition of each IFV subtype showed the specific adsorption of H1N1 human IFV as described above (see Supplementary Figure S1). In addition, the FET with the Siaα2,3′Lac-immobilized surface showed a high specificity for H5N1. Moreover, the Δ*V*_g_ caused by the addition of H5N1 to the Siaα2,3′Lac-immobilized FET was higher than that seen for the Siaα2,6′Lac-immobilized FET following the addition of H1N1. This is likely because the density of the immobilized Siaα2,3′Lac molecules is higher due to its linear glycan topology^13^. Furthermore, we measured the sensor responses at different concentration of IFV over the range from 10^0.5^ to 10^8.5^ TCID_50_/mL (50% tissue culture infectious dose) for each glycan-immobilized FET biosensor (Fig. 2). Note that the concentration of prepared viruses (the unit: TCID_50_/mL) was quantified by a measure of infectious virus titer. As expected, a correlation between the FET responses and IFV concentrations was clearly observed. The limit of detection (LOD) of the glycan-immobilized FET biosensor, calculated using 3σ of the standard deviation, was 10^0.5^ TCID_50_/mL, which is better than that for immunochromatography. Hence, these results suggest that the glycan-immobilized FET biosensor could discriminate between human and avian IFVs with both high sensitivity and specificity.

To understand how the viral particles attach to the glycan-immobilized sensing surface, we calculated and compared the experimentally-detected charge numbers attributed to the FET responses and the theoretical charge numbers of a single viral particle present in a charge detectable region of FET biosensing, Debye length. Note that a size of single whole viral particle is approximately 100 nm in diameter, whereas the Debye length in the environment under nearly physiological conditions, i.e. 1 × PBS, was 0.75 nm^22^. From the equation derived by Graham^23^, the charge density (σ_0_) attributed to the adsorbed IFV particles was calculated equal to be 9.4 × 10^−7^ C∙cm^−2^ at a response magnitude of 73.5 mV which was obtained after the addition of the IFV particles (10^8.5^ TCID_50_/mL H5N1 avian IFV) onto the Siaα2,3′Lac-immobilized FET biosensors, giving an electron density (*ρ*_e_) of 5.9 × 10^12^ cm^−2^. Since the density of adsorbed H5N1 IFV particles (*ρ*_IFV_) was estimated to be 6.9 × 10^8^ cm^−2^ by the AFM analysis, the experimentally-detected number of charges (*Z*_d_) was calculated to be −8.5 × 10^3^. On the other hand, theoretical number of charges on a single viral particle was calculated to be −2.9 × 10^4^ by counting up the charge numbers of all the units of the proteins, which are present on the surface of the particle′s envelope, i.e. the HA trimer, the NA tetramer, the M2 tetramer, and the phosphatide^24,25^ (see Supplementary Table S1). By taking into consideration both the experimentally-detected number of charges and the theoretical number of charges, a single viral particle appeared to be slightly-squashed, so that it attached to the glycan-immobilized surface with 29% of its viral surface area. The attached particles might exclude the PBS solution, which exists between the viral surface and the glycan-immobilized surface, suggesting that the Debye screening effect was decreased. Thus the charges of the viral particle that exist near the interface could be successfully detected by the glycan-immobilized FET biosensor under nearly physiological conditions.

### Optimization of the viscosity reduction treatment using L-cysteine ethyl ester for the direct detection of influenza virus in nasal mucus

**Figure 3:**
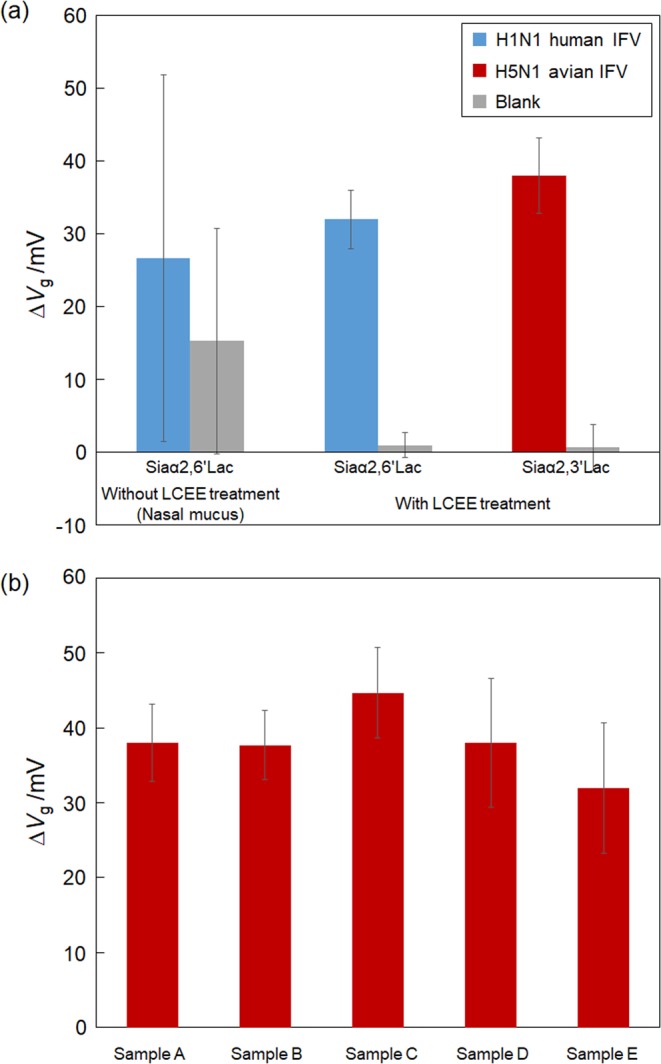
Effect of LCEE treatment on the FET responses to H1N1 IFV or avian H5N1 IFV. (**a**) Comparison of the FET responses to IFV samples with or without LCEE treatment. The concentrations of IFV particles added to the sensing surface were 10^7.5^ TCID_50_/mL for the sample without LCEE treatment, and 10^6.5^ TCID_50_/mL for the sample with LCEE treatment. The samples were prepared by mixing a suspension of the IFVs with nasal mucus. Error bars represent standard deviation (N = 3). (**b**) Spike test for the detection of influenza virus suspended in nasal mucus using LCEE treatment. The concentration of H5N1 avian influenza virus was 10^6.5^ TCID_50_/mL. Error bars represent standard deviation (N = 3).

We next attempted to directly detect IFV particles present in a biological fluid, i.e. nasal mucus, using the glycan-immobilized FET biosensors. First, sensor responses after the incubation with the H1N1 human IFV sample suspended in a nasal mucus, collected from a healthy person, were obtained using the Siaα2,6′Lac-immobilized FET biosensor (Fig. 3a). The magnitude of the response obtained from the IFV in nasal mucus was decreased compared with that in buffer solution, presumably because diffusion of the IFV particles to the FET gate surface was inhibited by the high viscosity of the nasal mucus. Small magnitude responses (15 mV) to the mucus solution were observed because nonspecifically adsorbed proteins, such as mucin, lysozyme, IgG, and albumin^26^, which remained on the FET gate surface after rinsing with PBS, also affected the overall response. In addition, these nonspecifically adsorbed proteins, which remained on the glycan-immobilized surface, might inhibit the specific adsorption of IFV particles. A large standard deviation was therefore obtained for each experiment due to variability in the amount of residual mucin and contaminating proteins present in each measurement. To examine the effect of mucin on the sensing surface, we observed the Siaα2,6′Lac-immobilized surface, before and after the addition of each sample, using AFM. The AFM images showed that the glycan-immobilized surface became undulating and spiky, suggesting that residual mucin molecules remained on the surface (see Supplementary Figure S3), whereas no granular objects that could be attributed to IFV particles were observed. Thus, the adsorption of IFV particles might be suppressed as a result of the increase in viscosity, as well as the presence of mucin, and other contaminating molecules, on the glycan-immobilized surface.

To reduce the viscosity of the nasal mucus to allow for the glycan-immobilized FET to give an adequate performance, we optimized a process whereby treatment with LCEE used to reduce viscosity. LCEE is a mucoactive agent that can break the disulfide bonds in mucin structures^18^. To optimize the LCEE concentration, we investigated the relationship between the responses after the addition of H1N1 human IFV particles (concentration of 10^6.5^ TCID_50_/mL) using the addition of different LCEE concentrations ranging from 1 mg/mL to 100 mg/mL. The maximum response was obtained when the sample was treated with 10 mg/mL LCEE solution (see Supplementary Figure S4). In case of 1 mg/mL LCEE solution, since the viscosity reduction was insufficient, the amount of specifically-adsorbed IFV particles was relatively small, whereas signals decreased as the LCEE concentration increased from 50 to 100 mg/mL, presumably as a result of the fact that the decrease in the pH of the LCEE solution might influence the properties of the viral surface protein HAs. It has been shown that the higher-order structure present in HA becomes denatured at pH 4.6–6.0^27^, reducing the ability of HA to recognize glycan. Additionally, the negative charge of the mucin surface decreased at low pH values, resulting in an increase in the viscosity of mucin due to the lower electrostatic interaction between mucin molecules^28^. It should be noted that our preliminary experiment revealed that the solution pH decreased as the LCEE concentration increased (see Supplementary information). In addition, the AFM images showed that objects with a height of approximately 100 nm were observed on the Siaα2,6′Lac-immobilized surface after the addition of H1N1 IFV particles, whereas no objects were observed on the Siaα2,6′Lac-immobilized surface alone (see Supplementary Figure S5). As a result, 10 mg/mL LCEE was determined to be the optimal concentration for the direct detection of IFV in nasal mucus. By treating the mucus sample with the LCEE solution, the responses obtained were larger and clearer than for the untreated sample (Fig. 3a), suggesting that the reduction in viscosity caused by LCEE treatment improved the interaction efficiency between IFV particles and glycans immobilized on the sensing surface. To determine if this viscosity reduction treatment, using the LCEE solution, could be used in general, sensor responses were obtained from the IFV samples suspended in the nasal mucus obtained from various people (Fig. 3b). Almost same magnitudes in the signals were obtained from each sample following the addition of H5N1 avian IFV containing nasal mucus treated with 10 mg/mL LCEE. These data suggest that LCEE treatment could be used to treat any biological samples collected from people or birds, and allow the glycan-immobilized FET biosensor to specifically detect IFV particles.

### Evaluation of the dual-channel glycan-immobilized FET biosensor for the detection of influenza virus at the source of outbreaks

**Figure 4:**
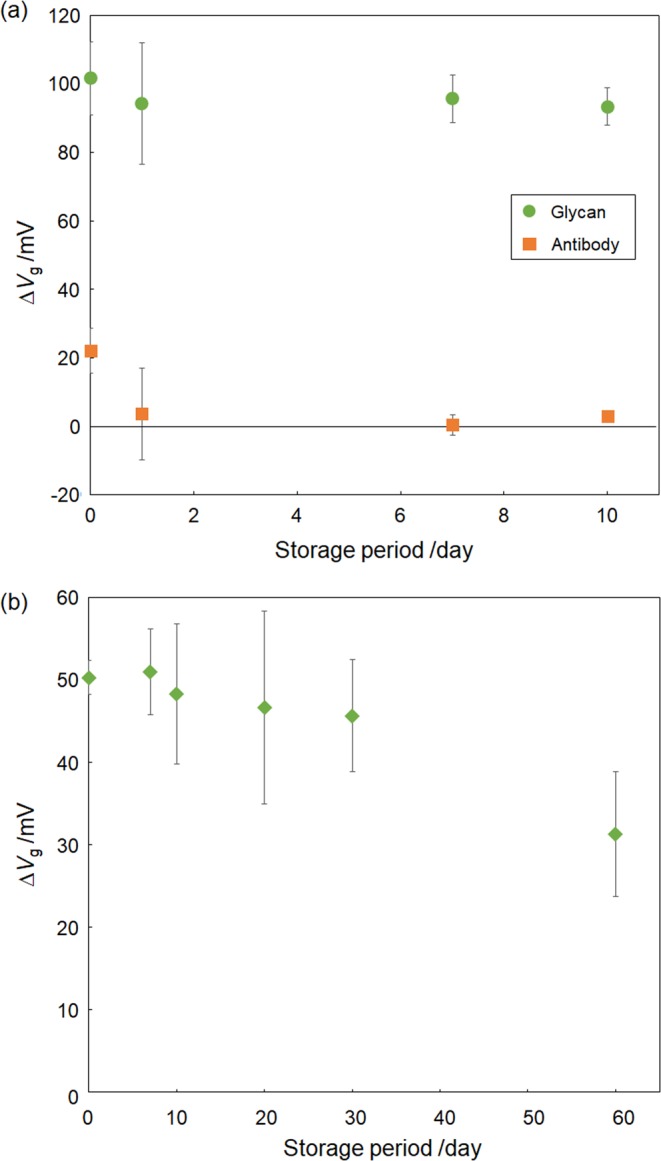
Evaluation of the storage stability of the glycan-immobilized FET biosensors. (**a**) Comparison of the FET responses obtained over time using either glycan-immobilized or antibody-immobilized FETs for the detection of the viral surface protein hemagglutinin (A/H1N1/New Caledonia/20/1999). The concentration of hemagglutinin was 1 μg/mL. Error bars represent standard deviation (N = 3). (**b**) Long-term stability of the Siaα2,6′Lac-immobilized FET biosensor for the detection of influenza virus. The concentration of human H1N1 IFV was 10^8.5^ TCID_50_/mL. Error bars represent standard deviation (N = 3).

**Figure 5:**
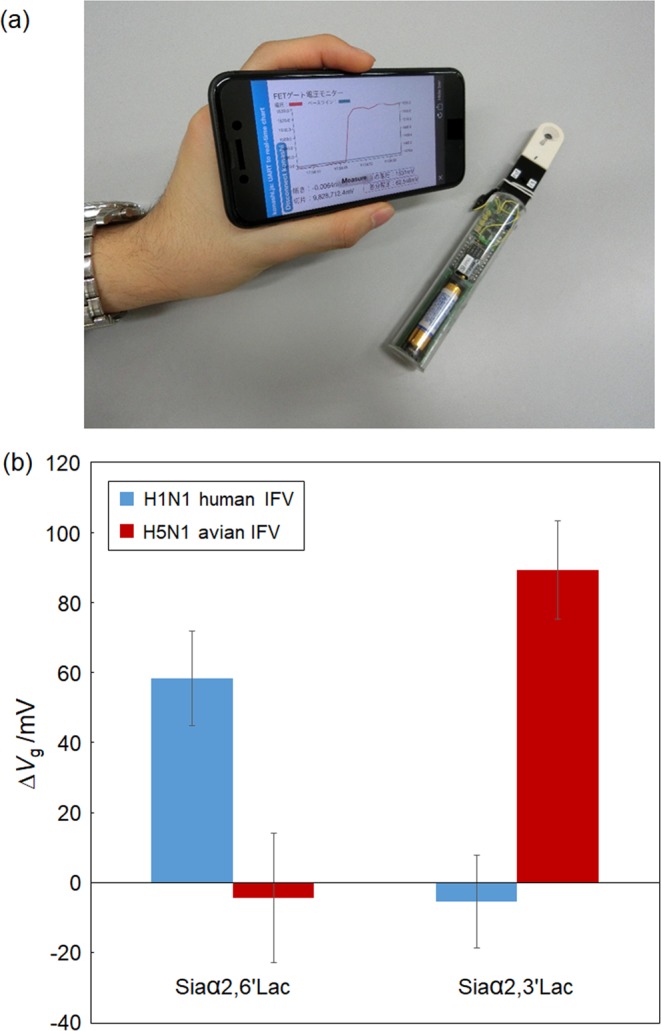
Portable biosensing system based using a Bluetooth connection between a smartphone and the FET biosensor. (**a**) Picture of the portable biosensing system. (**b**) FET responses obtained by the system following the addition of influenza virus. The concentration of human H1N1 IFV or avian H5N1 IFV were 10^6.5^ TCID_50_/mL. Error bars represent the standard deviation (N = 3).

To examine the storage stability of the glycan-immobilized FET biosensor, the sensor responses of the biosensor, which was stored for different fixed periods of time, were evaluated by comparing the magnitude of the responses caused by either the specific adsorption of the HA protein or IFV particles. First, we prepared both glycan-immobilized FET biosensors and antibody-immobilized FET biosensors, both of which were stored in a desiccator for different periods of time. Following this, we compared the changes in responses caused by the addition of 1 μg/mL H1 HA (A/H1N1/New Caledonia/20/1999), which is present on the surface of H1N1 human IFV. As shown in Fig. 4a, the responses obtained from the glycan-immobilized FET biosensors were stable, even after 10-days of storage, with their relative stability being equal at around 92%. In contrast, the responses obtained from the antibody-immobilized FETs decreased after one day of storage (the relative stability decreased to 17%). These results suggest that glycan molecules maintain their chemical structure even under a dry environment, although this same environment was detrimental to the antibody. This allows the glycan molecules to maintain their ability to recognize their target molecules, whereas the antibody molecules lose their ability to recognize their target antigen presumably because of their denaturation^29,30^. It should be noted that the magnitude of the response of the glycan-immobilized FETs was larger than that of antibody-immobilized FETs on the first day (0 day) because the small receptors, glycans, takes an advantage of effectively using the charge detectable region compared with big antibody molecules^13^. Thus, the glycan-immobilized FET biosensors are suggested to be more stable and sensitive than antibody-immobilized FET biosensors. Subsequently, the long term stability of the glycan-immobilized FET biosensor was then examined by assessing its ability to directly detect 10^8.5^ TCID_50_/mL H1N1 human IFV over time. After 30-days of storage, the glycan-immobilized FET biosensors showed relatively stable responses (relative stability of 91%) compared with freshly prepared biosensors (Fig. 4b). Even after 60-days of storage, significant responses were still observed, although the magnitudes were measurably decreased. These results suggest that the glycan-immobilized FET biosensors could be stored longer in preparation for the imminent appearance of pandemic influenza. To assess the feasibility of remote biosensing, control of the FET-based biosensing system using a smartphone device was developed based on our previously-described technology^31^. Because the biosensor should be easily carried around, and used when and where a novel influenza breaks out, the biosensor system was connected to the smartphone via a Bluetooth connection (Fig. 5a). As shown in Fig. 5b, the system, which could be remotely controlled by a smartphone, correctly provided sensing data obtained after the addition of the IFVs present in a nasal sample, suggesting that the system can quickly transfer electrical measurement results to the smartphone to enable rapid information sharing.

**Figure 6:**
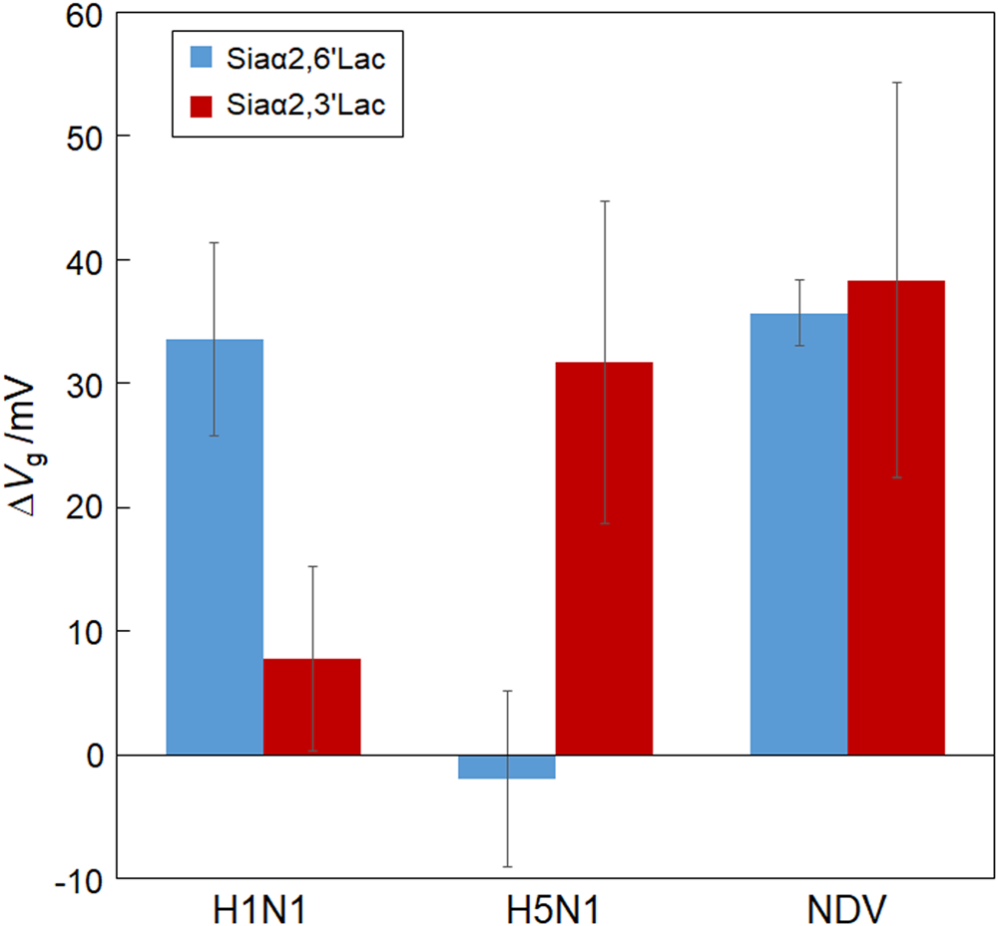
Discrimination of three kinds of viruses, namely, human influenza virus (H1N1), avian influenza virus (H5N1), and Newcastle disease virus (NDV), using a dual-channel, glycan immobilized, FET biosensor. Two types of glycans, namely Siaα2,6′Lac and Siaα2,3′Lac, were immobilized on each gate surface of the FET biosensor. Error bars represent standard deviation (N = 3).

To examine the possibility of the glycan-immobilized dual-channel FET biosensor being used to identify pandemic IFVs, the ability to directly detect three different kinds of viruses, namely H1N1 human IFV particles, H5N1 avian IFV particles, and NDV particles, spiked into nasal mucus, was investigated. Here we focused on the effectiveness of the biosensor with dual sensing surfaces separately modified with either human or avian glycan receptors in assessing the infectivity of new pandemic IFVs that could bind to both receptors. We assumed that the response at each of dual channels, depending on the binding affinity to each receptor, can be a measure of the infectivity of viruses. The FET biosensor used in this work possesses two gate surfaces in a chip, expecting that the infectivity of new pandemic IFVs can be judged by taking advantage of separately-immobilized Siaα2,6′Lac and Siaα2,3′Lac surfaces. In this study, the NDV particle was selected as a good model and substitute for a pandemic IFV particle because the NDV’s surface protein, hemagglutinin-neuraminidase, like a pandemic IFV’s surface protein, has the ability to recognize both α2,6 and α2,3 sialic acid-linkages^19,20^. For the identification of the pandemic IFV strain, two gate surfaces of the dual-channel FET biosensor were functionalized with Siaα2,6′Lac and Siaα2,3′Lac, respectively. Following this, clear responses corresponding to each viral addition were obtained when H1N1 human IFV particles, H5N1 avian IFV particles and NDV particles were added to the FET biosensor (Fig. 6). When the H1N1 human IFV particles or H5N1 avian IFV particles were allowed to react with the sensors, the particles were specifically captured by either of the glycan-immobilized channels. In contrast, interestingly, the results revealed that, in the case of the NDV particles, the sensor responses were clearly obtained from both channels. The results suggest that a dual-channel FET biosensor could be useful in discovering pandemic IFV particles by adapting a simple LCEE treatment to reduce mucus viscosity to allow for surveillance of novel influenza outbreaks.

## Conclusions

The direct detection of both human and avian IFV particles present in nasal mucus was demonstrated using a glycan-immobilized dual-channel FET biosensor aimed at allowing for the early identification of pandemic IFVs. The glycan-immobilized FET biosensor could detect human or avian IFV particles over a wide range of concentrations (between 10^0.5^ and 10^8.5^ TCID_50_/mL). Moreover, treatment with LCEE was useful in reducing the viscosity of the mucus samples, enabling the direct detection of IFV in nasal mucus. In addition, the glycan-immobilized FET sensor had good storage stability, because glycan molecules are chemically stable even in a dry environment. To examine the potential ability of the biosensor to detect pandemic IFVs, we successfully demonstrated the ability of glycan-immobilized, dual-channel, FET biosensor to detect the NDV, which is a prime model of a pandemic IFV. By using the portable biosensing system developed here, connected with a smartphone via Bluetooth technology, the spread of a pandemic IFV, which possess the binding ability to bind to both avian and human glycan receptors, could possibly be prevented at the early stages of its appearance.

## Methods

### Materials

The human and avian influenza virus (A/Hyogo/YS/2011 (H1N1) 2009pdm and A/duck/Hokkaido/Vac-3/2007 (H5N1)), Newcastle disease virus (NDV/duck/Hokkaido/204/2004), and PBS containing β-propiolactone were prepared at Hokkaido University (Sapporo, Japan). The IFV and NDV samples were inactivated by treatment with β-propiolactone and formalin, respectively. Influenza hemagglutinin (A/H1N1/New Caledonia/20/1999, recombinant, full-length, and maintaining the oligomeric structure of the protein) was purchased from Protein Sciences Co. (Meriden, CT, USA). The glycans, Siaα2,6′Lac and Siaα2,3′Lac, were purchased from Carbosynth Limited (Berkshire, UK). The antibody, anti-H1 (A/New Caledonia/20/1999), was purchased from Immune Technology Corp. (New York, USA). The self-assembled monolayer (SAM) reagent, 3-aminooxypropyltriethoxy-silane (AOPTES), was purchased from Medicinal Chemistry Pharmaceuticals, LLC (Sapporo, Japan). Acetic acid was purchased from Wako Pure Chemical Industries, Ltd. (Osaka, Japan). The surfactant Tween 20 and l-cysteine ethyl ester (LCEE) hydrochloride were purchased from Tokyo Chemical Industry Co., Ltd. (Tokyo, Japan), whereas all other chemicals were purchased from Kanto Chemical Co. Inc. (Tokyo, Japan). The n-type FETs, which were essentially based on our developed technology, were fabricated by Toppan Printing Co., Ltd. (Tokyo, Japan)^32,33^. Each FET chip consists of two channels, and its gate size was 10 μm (length) × 1000 μm (width). The device holder for the FET biosensor was fabricated by Yamamoto-MS Co., Ltd. (Tokyo, Japan). The Bluetooth biosensing system was developed by Symphodia Phil Co., Ltd. (Yamagata, Japan) and Toppan Printing Co., Ltd. The custom-ordered remote sensing system was composed of two parts; one was for the measurement and analysis of electrical characteristics of the FET biosensors controlled by an embedded microcontroller, and the other was for the wireless data transfer to smartphones. All proteins were used without further purification. Phosphate buffered saline (PBS) pH 7.4 was made in-house using 137 mM NaCl, 8.1 mM Na_2_HPO_4_·12H_2_O, 2.7 mM KCl, and 1.5 mM KH_2_PO_4_. Diluted PBS, (0.01 × PBS, pH 7.4), was prepared by diluting PBS with ultrapure water. All experiments handling IFVs and HA proteins were conducted in a laboratory rated at biosafety level 2 (BSL-2).

### Fabrication of glycan-immobilized FET

The glycan was immobilized to the FET gate surface thorough the SAM of AOPTES according to our previously proposed procedure^13^. Briefly, the surface of silicon dioxide, as a gate insulating film in the FET, was exposed to O_2_ plasma ashing (200 W for 1 min) to introduce hydroxyl groups onto the surface. Subsequently, the SAM was formed on the silicon dioxide surface by immersing it in 0.1% (v/v) AOPTES in toluene under an argon atmosphere (60 °C for 15 min). Following this, a glycan-containing solution (100 μM), prepared by dissolving either Siaα2,6′Lac or Siaα2,3′Lac in an acetic acid solution (The pH was controlled equal to 5.3), was allowed to react with the aminooxy moiety of the aminooxypropylsilane (AOPS)-modified surface (60 °C for 90 min), resulting in the fabrication of the glycan-immobilized FET biosensor. Note that the dual-channel FET biosensor consists of two transistors, both which were modified with different types of glycans, separately.

### Electric measurements

The gate voltage (*V*_g_) - drain current (*I*_d_) relationship of the glycan-immobilized FET was measured and used as the reference. Measurements were made in the dark using a System SourceMeter (2612 A, Keithley Instruments Inc., Ohio, USA) at room temperature in PBS (pH 7.4) by sweeping the *V*_g_ from −3.0 V to 0.5 V, and setting a constant *V*_d_ at 0.1 V. The reference electrode was Ag/AgCl. The IFV samples were allowed to react with the glycan-immobilized FETs for 10 min. IFV samples spiked in nasal mucus were obtained by mixing an IFV solution with nasal mucus collected by wiping the noses of five healthy people. Each sample was also treated with the LCEE solution to reduce mucus viscosity. After incubation, the residue was washed with PBS. The *V*_g_ - *I*_d_ characteristic of the FET biosensor, after incubation with IFV samples, was measured, and the threshold voltage shift (Δ*V*_g_) was calculated by comparing to the reference characteristics.

### Surface analyses using atomic force microscopy

Surface morphologies before and after target solution addition onto the FET surface were analyzed using AFM. Morphologic images of the gate surface of the FETs were investigated using a dynamic mode AFM (SPM-9600, Shimadzu Co., Kyoto, Japan). For measurement, a silicon cantilever (OMCL-AC240TSC2, Olympus Co., Tokyo, Japan, spring constant 2 N/m, resonance frequency 70 kHz) was used, with the observed image sizes being either 1 μm^2^ or 9 μm^2^ and containing 512 × 512 pixels. Roughness parameters were obtained from the AFM images.

## Supplementary information

Supplementary Information
