# Supplementary material for: Glycan-immobilized dual-channel field effect transistor biosensor for the rapid identification of pandemic influenza viral particles
Source: Sci Rep. 2019 Aug 12;9:11616. doi: 10.1038/s41598-019-48076-6 (PMC6691001; doi:10.1038/s41598-019-48076-6)
Supplement: Supplementary file 1 — Supplementary Information [file 41598_2019_48076_MOESM1_ESM.docx]

Supplementary Information

Glycan-immobilized dual-channel field effect transistor biosensor for the rapid identification of pandemic influenza viral particles

Sho Hideshima^1,†,^*****, Hiroki Hayashi^2^, Hiroshi Hinou^3^, Shunsuke Nambuya^2^, Shigeki Kuroiwa^1^, Takuya Nakanishi^1^, Toshiyuki Momma^1,2^, Shin-Ichiro Nishimura^3^, Yoshihiro Sakoda^4^, Tetsuya Osaka^1,2,^*****

*^1^ Research Organization for Nano & Life Innovation, Waseda University, 513 Waseda-tsurumaki-cho, Shinjuku-ku, Tokyo 162-0041, Japan*

*^2^ Graduate School of Advanced Science and Engineering, Waseda University, 3-4-1 Okubo, Shinjuku-ku, Tokyo 169-8555, Japan*

*^3^ Faculty of Advanced Life Science, Hokkaido University, N21, W11, Kita-ku, Sapporo-shi 001-0021, Hokkaido, Japan*

*^4^ Faculty of Veterinary Medicine, Hokkaido University, N18, W9, Kita-ku, Sapporo-shi 060-0818, Hokkaido, Japan*

*^†^ Current address: Research Initiative for Supra-Materials, Interdisciplinary Cluster for Cutting Edge, Shinshu University, 3-15-1 Tokida, Ueda-shi, Nagano 386-8567, Japan*

*** Corresponding Author:**

Tetsuya Osaka

osakatets@waseda.jp

Waseda University, Okubo 3-4-1, Shinjuku-ku, Tokyo 169-8555, Japan

TEL: +81-3-5286-3202; FAX: +81-3-3205-2074

**Observation of glycan-immobilized surfaces caused after the addition of IFVs by using atomic force microscopy**

To confirm the specificity of the glycan-immobilized surface to IFV particles, the surface morphology was observed by atomic force microscopy (AFM). The surface morphology of the Siaα2,6'Lac-immobilized surface was changed after incubation (10 min) with 10^8.5^ TCID_50_/mL H1N1 human IFV (A/H1N1pdm/Hyogo/YS/2011) (Figure S1a and b). The image shows that the size of the observed particles matches the diameter of the IFV’s grain (~100 nm). On the other hand, after the addition of 10^8.5^ TCID_50_/mL H5N1 avian IFV (A/H5N1/duck/Hokkaido/Vac-3/2007) the surface morphology and roughness were not significantly changed (Figure S1c), indicating that the Siaα2,6'Lac-immobilized surface cannot bind avian IFV. The hydrophilicity of the glycan moiety might also suppress hydrophobic interactions of non-specific contaminating substances. Thus, these results suggest that the Siaα2,6'Lac-immobilized surface can specifically bind human H1N1 IFV.


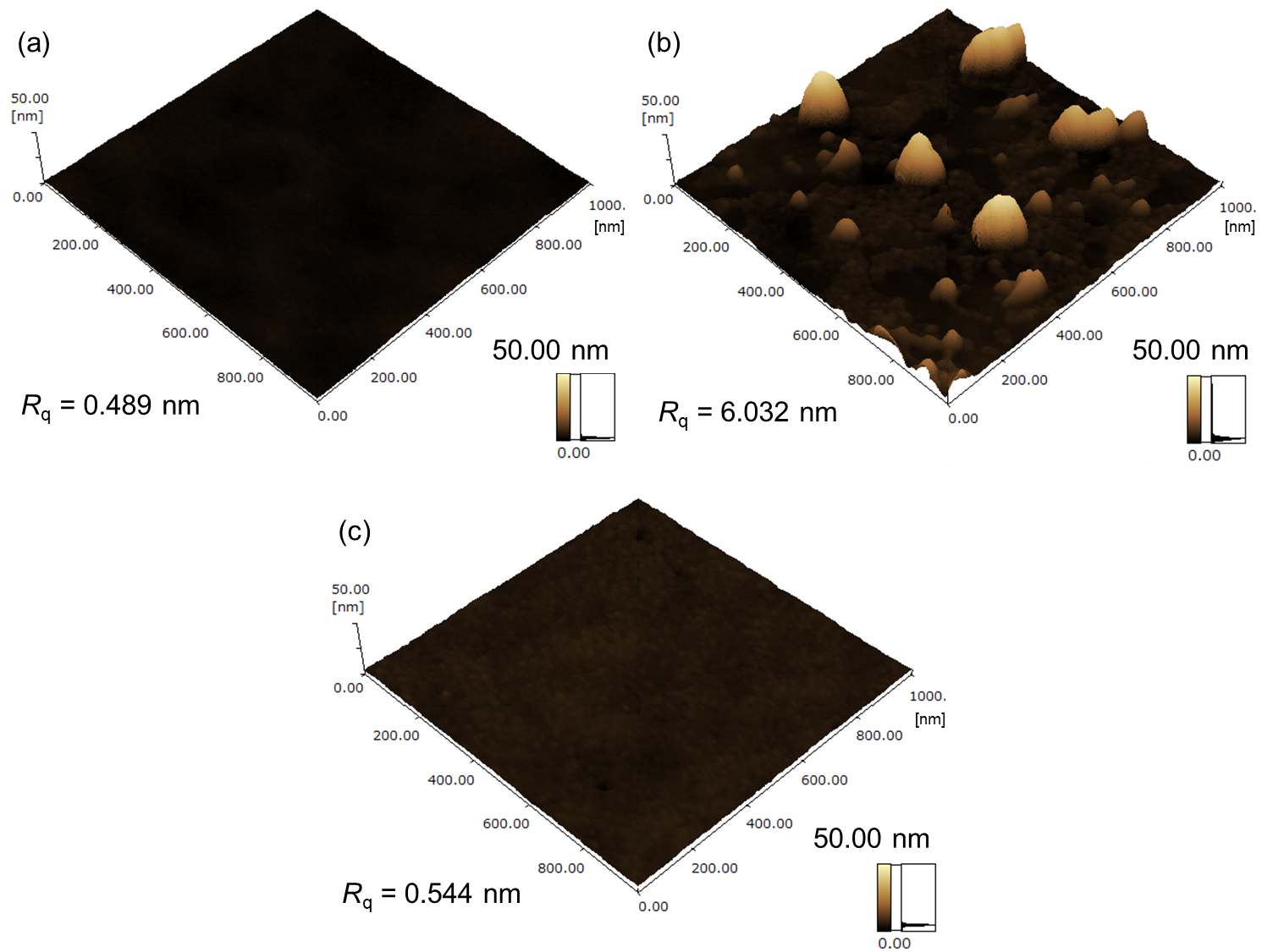


**Figure S1** Atomic force microscopic images of Siaα2,6'Lac-immobilized surfaces after the addition of H1N1 human and H5N1 avian IFVs. The images show the morphology of (a) Siaα2,6'Lac-immobilized surfaces, and the surfaces after the addition of (b) H1N1 human IFV and (c) H5N1 avian IFV. The scan size was 1 × 1 μm^2^. The AFM observation was made in air.

**Principle of the detection of whole IFV particles in a high ionic strength environment using FET biosensors**

The charge detectable region of FET biosensors, namely the Debye length, strongly depends on the ionic strength of the measurement environment. The Debye length is calculated to be 0.75 nm by using the Debye-Huckel equation^[1]^, when the target molecules are detected under a high ionic strength condition (1 × PBS, *I* = 165 mM). Thus, we determined how IFVs could be detected in 1 × PBS using FET biosensors, because the size of IFV particles, whose diameter is ca. 100 nm, is beyond the Debye length. Here, to investigate the detected partial region of the viral membrane, we calculated the number of negative charges per viral particle detected by the FET biosensor, and compared them with the number of charges on the envelope surface based on the theoretical viral structure.

When a significant response (73.5 mV) was observed after the addition of 10^8.5^ TCID_50_/mL H5N1 avian IFVs to Siaα2,3'Lac-immobilized FET biosensors, the charge density (σ_0_) detected by the FET biosensor was 9.4 × 10^-7^ C∙cm^-2^, calculated using the Graham equation ^[2]^ (eq. 1),

$$\sigma_{0}=\sqrt{8000IN_{A}\varepsilon_{o}\varepsilon_{w}kT}\sinh\frac{e\Delta V_{g}}{2kT} (eq. 1)$$

where *I* is the ionic strength of the buffer solution, N_A_ is the Avogadro constant, ε_0_ is the permittivity of a vacuum, ε_w_ is the permittivity of water at 25ºC, *k* is the Boltzmann constant, *T* is the absolute temperature in Kelvin, and e is the elementary charge. Following this, the number density of electron (ρ_e_) was shown to be 5.9 × 10^12^ cm^-2^ which was obtained by dividing σ_0_ by the elementary charge (*e* = 1.60 × 10^-19^ C). The density of the adsorbed H5N1 IFV particles (ρ_IFV_) on the surface was estimated to be 6.9 × 10^8^ cm^-2^ by counting the IFV particles observed by the AFM analysis (Figure S2). Thus, negative charge numbers, *Z*_d_, was found to be -8.5 × 10^3^ by calculating from the experimentally-obtained voltage shift. To obtain the theoretical charge numbers of the envelope of the IFV particle, we calculated the individual intrinsic charges of the four kinds of viral membrane molecules, hemagglutinin (HA), neuraminidase (NA), matrix protein 2 (M2) and a part of phosphatide (Table S1)^[3,4]^. The numbers of each protein per single IFV particle have been reported to 5.0 × 10^2^, 1.0 × 10^2^, and 10, for the HA trimer, the NA tetramer, and the M2 tetramer, respectively. The number of phosphatide molecules were estimated to be 8.4 × 10^4^ by taking into consideration both all of the numbers of the above-mentioned molecules that exist on the viral surface (HA, NA, and M2). The intrinsic charge numbers for each viral protein (HA, NA, M2), and phosphatide, at pH 7.4 were calculated equal to be -13.0, -6.25, -2.71, and -0.08, respectively, based on amino acid sequences (Table S1). Consequently, the theoretical charge numbers on single IFV particle possesses are -2.9 × 10^4^. Thus, by comparing the theoretical charge numbers and the experimentally-determined charge numbers, the results suggest that viral proteins, present in 29 % of the viral surface area, could attach to the glycan-immobilized surface. It should be noted that the adsorbed particles could remove the buffer solution present at the interface between the flexible particles ^[5,6]^ and the glycan-immobilized surface, suggesting that whole particles could be successfully detected in the high ionic strength environment due to the decrease of the Debye screening effect.


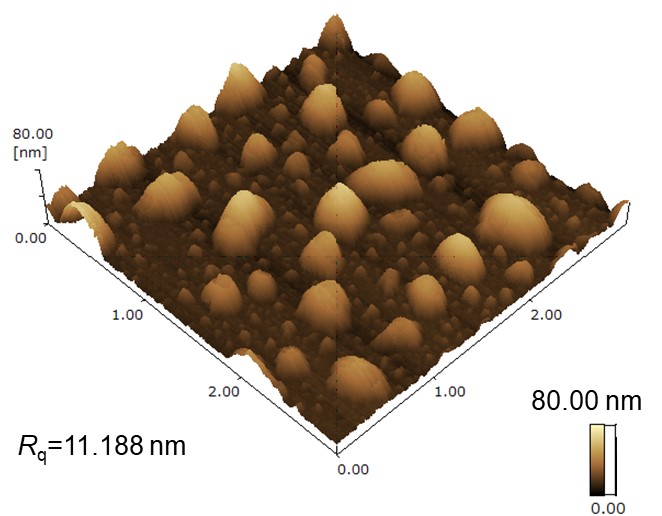


**Figure S2** Atomic force microscopic image of the Siaα2,3'Lac-immobilized surface after the addition of 10^8.5^ TCID_50_/mL H5N1 avian IFVs. The scan size was 3 × 3 μm^2^. The AFM observations were made in air. Z range = 80 nm.

| **Table S1** Number of units and charge numbers of molecules on viral membrane | | | | |
| --- | --- | --- | --- | --- |
|  | HA trimer | NA tetramer | M2 tetramer | Phosphatide |
| Number of units | 5 × 10^2^ | 1 × 10^2^ | 10 | 8.4 × 10^4^ |
| Charge per monomer | -13.0^*1^ | -6.25^*2^ | -2.71^*3^ | -0.08^[7]^ |
| Total charge | -2.0 × 10^4^ | -2.5 × 10^3^ | -1.1 × 10 | -7.0 × 10^3^ |

^*1^ UniProtKB - A7WRB7 (A7WRB7_9INFA), ^*2^ UniProtKB - A7WRC1 (A7WRC1_9INFA),

^*3^ UniProtKB - A7WRC4 (A7WRC4_9INFA)

**Atomic force microscopy observation of glycan-immobilized surface after the addition of the untreated mucus samples.**

Figure S3 shows the surface morphology of the glycan-immobilized surfaces caused by the immersion in an untreated mucus sample containing IFV particles. IFV particles were clearly not observed due to the influence of the mucin molecules, a major component glycoprotein in mucus (Figure S3a). Mucin molecules increase viscosity due to the formation of long-chain structures by disulfide bonds ^[8]^, resulting in an inhibition of the specific adsorption of IFVs to the glycan-immobilized surface. Moreover, the value of surface roughness (*R*_q_) increased from 0.489 nm (Figure S1a) to 0.656 nm (Figure S3b) as result of the nonspecific adsorption of contaminating proteins because the mucus sample had high viscosity and remained on the glycan-immobilized surface even after washing with the buffered solution. These results suggest that the mucus sample should be treated with a mucoactive agent in order to obtain FET signals from IFV particles.


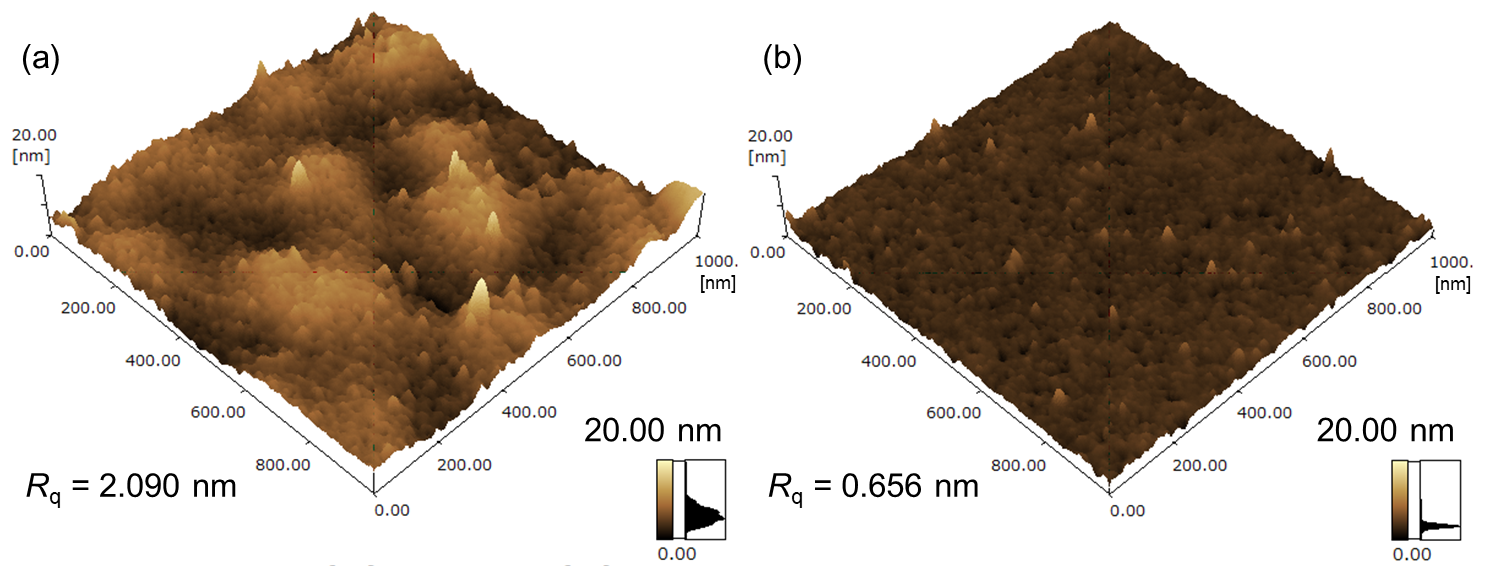


**Figure S3** Atomic force microscopic images of Siaα2,6'Lac-immobilized surfaces after addition of untreated mucin samples. After the addition of (a) untreated nasal mucus samples containing H1N1 human IFV and (b) untreated nasal mucus. The scan size was 1 × 1 μm^2^. The AFM observations were made in air. Z range = 20 nm.

**Optimization of the LCEE concentration used to reduce the viscosity of mucus samples**

To optimize the viscosity reduction of mucus samples using LCEE, we examined the FET responses caused by specific adsorption of H1N1 IFV particle (10^6.5^ TCID_50_/mL) on Siaα2,6'Lac-immobilized surface in samples treated with different LCEE concentrations. The magnitude of the specific adsorption of the IFV particles reached its maximum value at 10 mg/mL LCEE (Figure S4). The increase in the magnitude of ∆*V*_g_ with increasing LCEE concentrations, from 1 mg/mL to 10 mg/mL, is assumed to be associated with a decrease in the viscosity of the mucus samples. However, the signals decreased as the LCEE concentration further increases from 10 mg/mL to 100 mg/mL, which is assumed to be related a decrease in the binding ability of the viral surface protein, hemagglutinin (HA), because the HA molecule will denature as the pH of LCEE solution become lower ^[9]^ (pH 5.86 at 10 mg/mL LCEE and pH 4.05 at 100 mg/mL LCEE). Overall, 10 mg/mL LCEE was determined to be the optimal concentration for the viscosity reduction of the mucus sample.


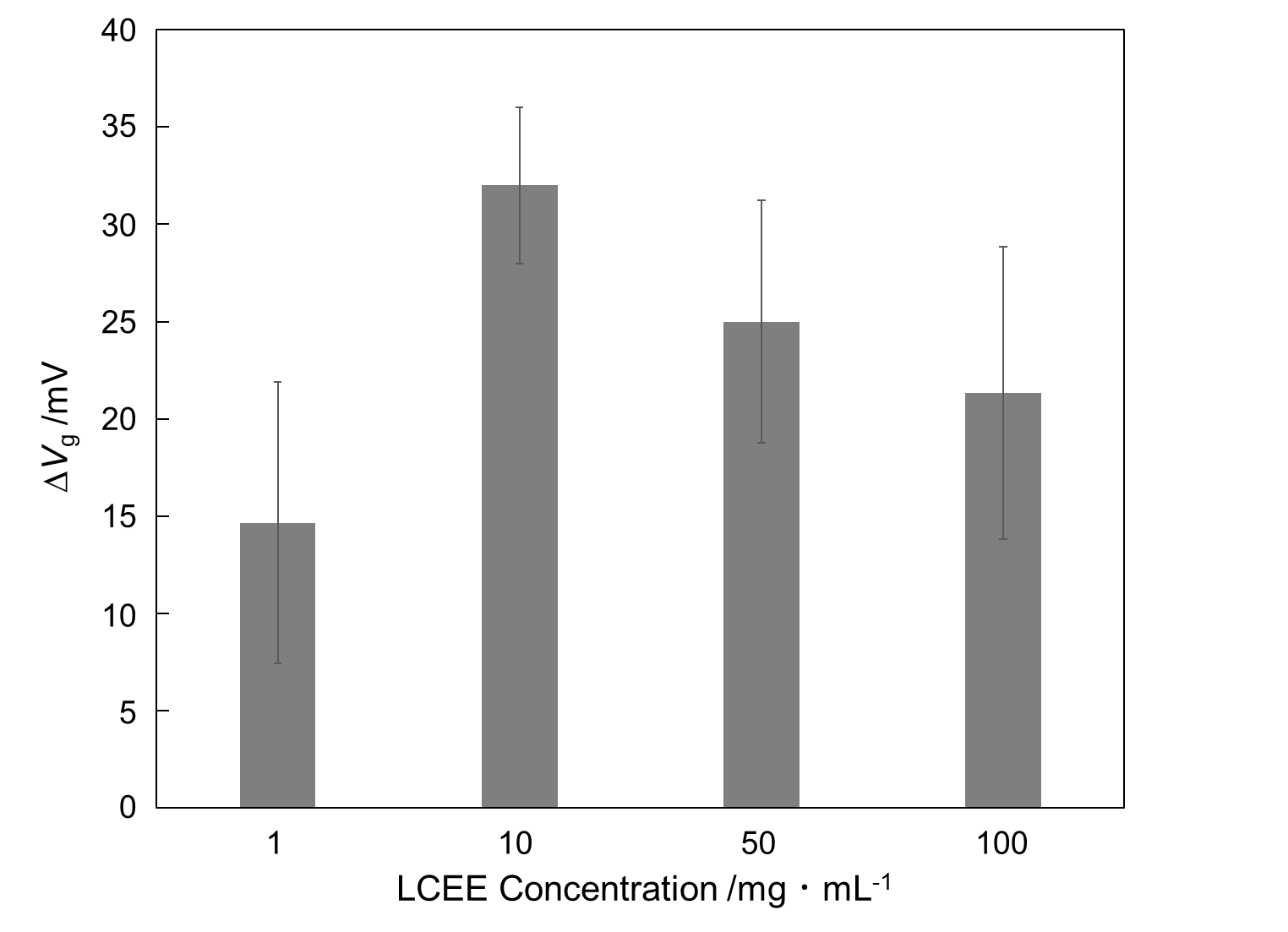


**Figure S4** Relationship between the LCEE concentration used for viscosity reduction and the magnitude of the FET response caused by the addition of IFV in nasal mucus. The IFV concentration was 10^6.5^ TCID_50_/mL.

**Atomic force microscopy observation of glycan-immobilized surfaces after the addition of mucus samples treated with LCEE**

To investigate the specificity of the glycan-immobilized surfaces to IFV particles in nasal mucus after treatment with 10 mg/mL LCEE, the Siaα2,6'Lac-immobilized surfaces were observed by AFM after the addition of H1N1 human IFV or H5N1 avian IFV. Grains were observed after the addition of H1N1 human IFVs to the Siaα2,6'Lac-immobilized surface (Figure S5a). On the other hand, no significant changes were observed after the addition of H5N1 avian IFVs (Figure S5b), suggesting that the glycan-immobilized surfaces can discriminate between the IFV subtypes. In addition, LCEE treatment could be useful in increasing the diffusion of IFV particles in the mucus samples.


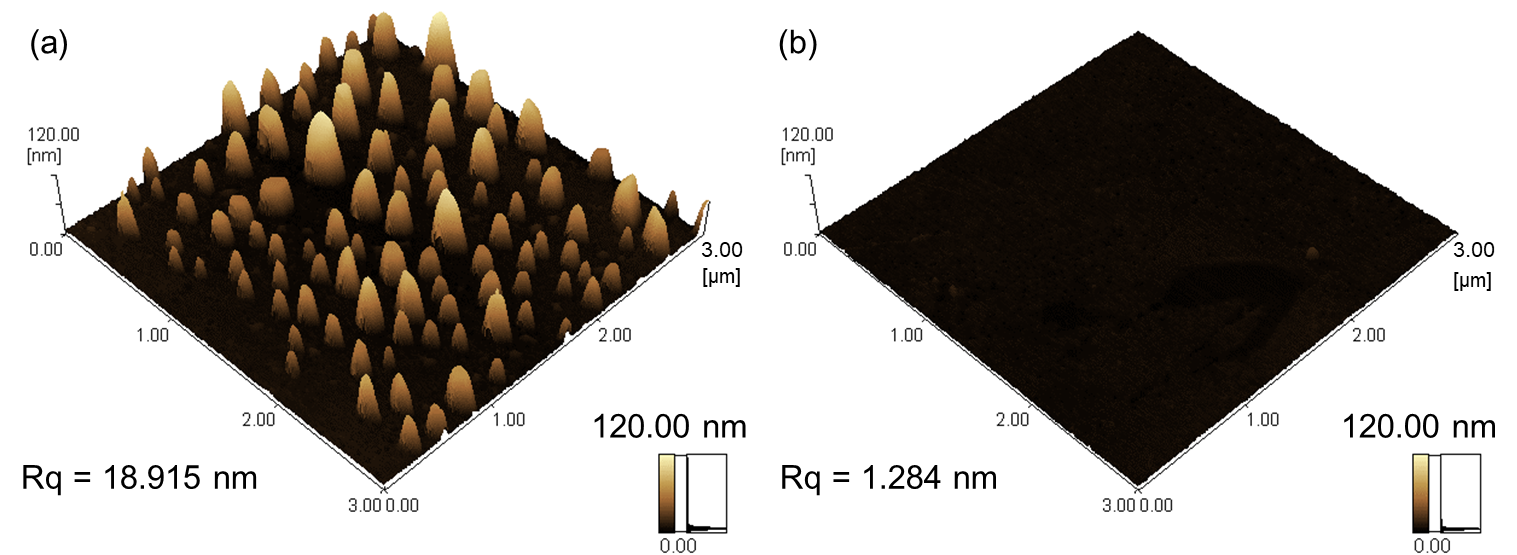


**Figure S5** Atomic force microscopic images of the Siaα2,6'Lac-immobilized surface after addition of nasal mucus containing influenza virus treated with 10 mg/mL LCEE. The images show the surface morphology caused by the addition of (a) H1N1 human IFV and (b) H5N1 avian IFV in nasal mucus.

**References**

1. Crow, D. R. *Principles and Applications of Electrochemistry*, FL, p. 259, (Taylor & Francis, 1994).
2. Allen J. Bard, L. R. F. *Electrochemical methods, fundamentals and applications*. *Journal of Chemical Education* (JOHN WILEY & SONS INC., 2000).
3. Murti, K. G. & Webster, R. G. Distribution of hemagglutinin and neuraminidase on influenza virions as revealed by immunoelectron microscopy. *Virology* **149**, 36–43 (1986).
4. Zebedee, S. L. & Lamb, R. A. Influenza A virus M2 protein: monoclonal antibody restriction of virus growth and detection of M2 in virions. *J. Virol.* **62**, 2762–72 (1988).
5. Harrison, S. C. Viral membrane fusion. *Nat. Struct. Mol. Biol.*, **15**, 7, 690-698 (2008).
6. Schaap, I. A., Eghiaian, F., des Georges, A. & Veigel, C. Effect of Envelope Proteins on the Mechanical Properties of Influenza Virus. *J. Biol. Chem.*, **287**, 49, 41078-41088, (2012).
7. Tanford, C. The Hydrophobic Effect: Formation of Micelles and Biological Membranes, Second edition, p.109, (Wiley, New York, 1980).
8. Bansil, R. & Turner, B. S. Mucin structure, aggregation, physiological functions and biomedical applications. *Curr. Opin. Colloid Interface Sci.* **11**, 164–170 (2006).
9. Scholtissek, C. Stability of infectious influenza A viruses at low pH and at elevated temperature. *Vaccine* **3**, 215–218 (1985).
